# Efficient and versatile CRISPR engineering of human neurons in culture to model neurological disorders

**DOI:** 10.12688/wellcomeopenres.10011.1

**Published:** 2016-11-15

**Authors:** Ruth R. Shah, Justyna Cholewa-Waclaw, Faith C.J. Davies, Katie M. Paton, Ronan Chaligne, Edith Heard, Catherine M. Abbott, Adrian P. Bird

**Affiliations:** 1 Wellcome Trust Centre for Cell Biology, University of Edinburgh, Edinburgh, UK; 2 Centre for Genomic and Experimental Medicine, MRC Institute of Genetics and Molecular Medicine, University of Edinburgh, Western General Hospital, Edinburgh, UK; 3 Centre de Recherche, Institut Curie, Centre National de la Recherche Scientifique, Unité Mixte de Recherche 3215, Institut National de la Santé et de la Recherche Médicale U934, Paris, France

**Keywords:** CRISPR, LUHMES, human, neurons, neurological, Cas9, MeCP2, EEF1A2

## Abstract

The recent identification of multiple new genetic causes of neurological disorders highlights the need for model systems that give experimental access to the underlying biology. In particular, the ability to couple disease-causing mutations with human neuronal differentiation systems would be beneficial. Gene targeting is a well-known approach for dissecting gene function, but low rates of homologous recombination in somatic cells (including neuronal cells) have traditionally impeded the development of robust cellular models of neurological disorders. Recently, however, CRISPR/Cas9 gene editing technologies have expanded the number of systems within which gene targeting is possible. Here we adopt as a model system LUHMES cells, a commercially available diploid human female mesencephalic cell line that differentiates into homogeneous mature neurons in 1-2 weeks. We describe optimised methods for transfection and selection of neuronal progenitor cells carrying targeted genomic alterations using CRISPR/Cas9 technology. By targeting the endogenous X-linked *MECP2* locus, we introduced four independent missense mutations that cause the autism spectrum disorder Rett syndrome and observed the desired genetic structure in 3-26% of selected clones, including gene targeting of the inactive X chromosome. Similar efficiencies were achieved by introducing neurodevelopmental disorder-causing mutations at the autosomal *EEF1A2* locus on chromosome 20. Our results indicate that efficiency of genetic “knock-in” is determined by the location of the mutation within the donor DNA molecule. Furthermore, we successfully introduced an mCherry tag at the *MECP2* locus to yield a fusion protein, demonstrating that larger insertions are also straightforward in this system. We suggest that our optimised methods for altering the genome of LUHMES cells make them an attractive model for the study of neurogenetic disorders.

## Introduction

The advent of technologies that introduce targeted mutations into the genome has dramatically changed the way in which genetic diseases can be modelled and studied. The most recent development in the genome editing field, the clustered regularly interspaced short palindromic repeats (CRISPR) and CRISPR associated 9 (Cas9) system, has proven to be extremely successful, due in part to its ease of use and efficient implementation in a variety of cell lines ( Cong *et al.*, 2013; Jinek *et al.*, 2013; Liang *et al.*, 2015) and model organisms ( Friedland *et al.*, 2013; Gratz *et al.*, 2013; Hai *et al.*, 2014; Hwang *et al.*, 2013; Jiang *et al.*, 2013; Li *et al.*, 2013; Niu *et al.*, 2014; Wang *et al.*, 2013). The coupling of CRISPR gene editing technology with human induced pluripotent stem cells (iPSCs) has rapidly expanded the number of neurological disorders that can be modelled in a human neuronal background and is allowing researchers to probe the underlying molecular mechanisms in unprecedented detail ( Howden *et al.*, 2016; Merkert & Martin, 2016; Mungenast *et al.*, 2016). In particular, the ability to genetically modify a hiPSC line to create isogenic cell lines, which are genetically identical (bar the disease causing mutation), and differentiate these into neurons for phenotypic analysis is extremely powerful. However, despite advances in hiPSC culture and neuronal differentiation protocols, there are still some limitations to this strategy. One obstacle is the large variability of clonal iPS cell lines when they are derived, which can have negative downstream effects on CRISPR targeting efficiency, single cell cloning and particularly on phenotypic outcomes. Furthermore, there is still debate as to the robustness of the epigenome in iPSCs after reprogramming ( Kim *et al.*, 2010; Ohi *et al.*, 2011).

Alternative human neuronal progenitor cell lines are available including the SH-SY5Y line and neural stem cells derived from fetal human brain or human embryonic stem cells. Yet each of these models has drawbacks. SH-5YSY cells are a neuroblastoma cell line with multiple chromosomal duplications and deletions ( Krishna *et al.*, 2014) and neural stem cells take a long time to mature during the differentiation process, expressing markers specific for neuronal progenitors for at least four weeks ( Shin & Vemuri, 2010; Sun *et al.*, 2008; Tong *et al.*, 2016). The LUHMES neuronal progenitor cell line is a recent alternative that is proving to be highly useful in the neuroscience field ( Lotharius *et al.*, 2002). These female “pre-neuronal” cells are forced to proliferate in an immature state by expression of the retroviral element v-myc ( Hoshimaru *et al.*, 1996). V-myc expression is under the control of tetracycline so by simple administration of the drug to cell culture medium, LUHMES cells undergo a rapid and robust differentiation into a homogeneous population of electrically active, post-mitotic, mature dopaminergic neurons in just 1 week ( Lotharius *et al.*, 2002). The resulting neurons have thus far been used to model Parkinson’s disease ( Lotharius *et al.*, 2005; Xiang *et al.*, 2013), for cytotoxicity assays ( Tong *et al.*, 2016) and for technology development ( Dinh *et al.*, 2013; Hughes *et al.*, 2014; Ilieva *et al.*, 2013).

In order to make this cell line more widely applicable for the neuroscience field, it would be beneficial to routinely genetically modify LUHMES cells to create a variety of cell lines for disease modelling and drug-screening purposes. Historically genetic manipulation via homology-directed repair (HDR) of somatic cells has been difficult, with the most successful approaches involving rAAV-delivered homology arms to produce targeting efficiencies of ~1% ( Porteus & Baltimore, 2003; Porteus *et al.*, 2003; Russel & Hirata, 1998). With the advent of CRISPR technologies, HDR targeting efficiencies in somatic cells has increased somewhat, although to different extents in different systems, for example 1.3% in primary neonatal fibroblasts ( Lin *et al.*, 2014), 1.8% *in vivo* by AAV delivery to mouse lung tissue ( Platt *et al.*, 2014) and 17% in T cells using Cas9 ribonucleoprotein complexes ( Schumann *et al.*, 2015).

Here we describe a robust and reproducible method for the efficient transfection of LUHMES cells, and demonstrate various ways in which this cell line can be genetically manipulated using CRISPR engineering to create human models for the study of neurological disorders.

## Results

### Transfection of proliferating LUHMES cells using Nucleofection

**Figure 1.**
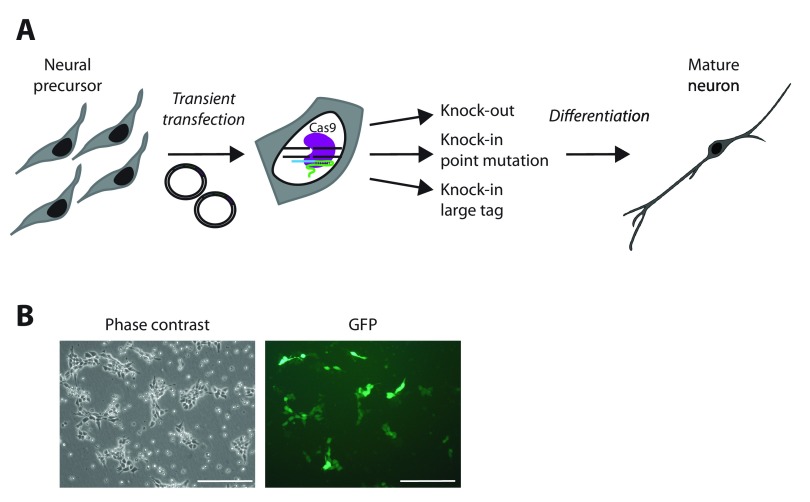
Nucleofection for efficient transfection of LUHMES cells. ( **A**) The aims of this study are to develop methods for genetic manipulation of the LUHMES pre-neuronal cell line, which in itself requires methods of transient transfection to be developed. After successful genetic modifications have been confirmed, LUHMES cells can then be differentiated into mature neurons for study. ( **B**) Representative images of plasmid transfection of LUHMES cells using program D33 with a Nucleofector device. Pictures were taken 47 hours post-Nucleofection. Scale bar is 200 μm.

In this study we sought to edit the endogenous genome of the pre-neuronal somatic LUHMES cell line in three ways: i) by disrupting a target gene; ii) by introducing discrete mutations into the protein coding region; iii) by adding a relatively large protein tag to generate a fusion protein ( Figure 1A). Karyotyping confirmed that LUHMES cells have a normal diploid chromosome complement ( Supplementary Figure 1A) and RNA FISH demonstrated X inactivation to be established in the pre-neuronal cells prior to differentiation ( Supplementary Figure 1B). As a first step in editing the genome, a reproducible method of plasmid transfection needed to be established for these cells. LUHMES cells have proven to be difficult to transfect ( Schildknecht *et al.*, 2013) and as a result previous studies relied on lentiviruses. In our hands transient transfection methods such as electroporation, Lipofectamine-2000, Neon transfection and JetPrime Polyplus all resulted in cell death or extremely low levels of transfection (Ruth R. Shah, Justine Cholewa-Waclaw, and Adrian Bird, unpublished report). Nucleofection has previously proven to be successful, but only after differentiating the cells for 2 days prior to trypsinisation and Nucleofection ( Schildknecht *et al.*, 2013). For the generation of genetically modified cell lines, this protocol is undesirable as any transfected cells will be immediately differentiated into post-mitotic neurons, and no stock of proliferating, genetically modified cells remains. In order to optimise Nucleofection conditions in proliferating LUHMES neuronal progenitor cells, the Amaxa Basic Nucleofector Kit for primary neurons was used with 20 different Nucleofection programs to find the optimal balance between transfection efficiency and cell viability ( Supplementary Figure 1C). Program D33 reproducibly yielded transfection efficiencies of 25–30%, as judged by the number of GFP-positive cells in the population ( Figure 1B). Of note, transfection of plasmids that have been prepared in an endotoxin-free environment resulted in increased cell viability, but purification of plasmids by ethanol precipitation did not improve this ( Supplementary Figure 1E+F). In this way we achieved efficient and reproducible transfection of proliferating LUHMES cells using exogenous plasmids.

### Generation of a MeCP2 knock-out cell line

**Figure 2.**
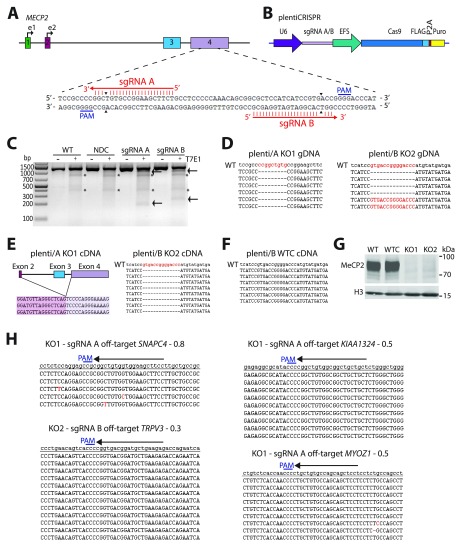
Generation of MeCP2 knock-out LUHMES cell lines. ( **A**) Schematic representation of the *MECP2* locus with the targeting sequence of sgRNA A and sgRNA B labelled. Arrowheads indicate sites of double-strand break. ( **B**) Schematic representation of the plasmid used to deliver Cas9 and sgRNA to LUHMES cells. ( **C**) T7E1 assay of sgRNA A and sgRNA B. WT: non-transfected wild-type cells. NDC: wild-type cells Nucleofected without any DNA. Asterisks indicate non-specific bands. Arrows indicate bands specific to samples containing Cas9 and sgRNA. ( **D**) Sequencing of genomic DNA from two LUHMES single-cell clones. Wild-type genomic DNA is in lower case at the top. ( **E**) Sequencing of cDNA from two LUHMES single-cell clones. Wild-type cDNA is in lower case at the top. ( **F**) Sequencing of genomic DNA from a single LUHMES unedited clone that was transfected with Cas9 and sgRNA B. Wild-type gDNA is in lower case at the top. ( **G**) Western blot of MeCP2 protein and Histone H3 loading control from wild-type cells (WT), wild-type cells that went through the Nucleofection and cloning process (WTC), KO1 and KO2 cell lines. ( **H**) Sequencing of sgRNA off-target sites in KO1 and KO2 cell lines. Numbers next to each locus name indicate the off-target score as determined by crispr.mit.edu.

We next tested the ability of CRISPR/Cas9 to generate a knock-out LUHMES cell line. For this the *MECP2* locus was chosen. The MeCP2 protein is highly expressed in neurons ( Shahbazian *et al.*, 2002; Skene *et al.*, 2010) and mutations within this protein lead to the autism-spectrum disorder Rett syndrome ( Amir *et al.*, 1999). Multiple mouse models of Rett syndrome have been developed, including mice containing Rett syndrome-causing point mutations ( Brown *et al.*, 2016) as well as knock-out alleles ( Chen *et al.*, 2001; Guy *et al.*, 2001). The *MECP2* gene has four exons, with different isoforms being expressed from exons 1 and 2. As exon 3 is the first shared exon among all isoforms, this was chosen for targeting in order to ablate all MeCP2 protein isoforms. Two sgRNAs were designed within exon 3 ( Figure 2A) and were individually cloned into a plasmid that also encodes Cas9 and a puromycin resistance gene ( Figure 2B) ( Sanjana *et al.*, 2014). LUHMES cells were Nucleofected ( Supplementary Figure 2A) and after selecting for positively transfected cells using puromycin resistance both sgRNAs were confirmed to be functional by the T7E1 assay ( Figure 2C) and single-cell colonies were established by serial dilution in 96-well plates. The genomic DNA from single cell colonies was extracted and sequenced in order to identify potential positive KO clones. The genomic DNA sequencing from two different cell lines is shown in Figure 2D, KO1 has a homozygous deletion of 9bp whereas KO2 has a heterozygous deletion of 14bp, with the second allele being unaltered. As *MECP2* resides on the X chromosome and LUHMES cells are female cells with one X chromosome already in the inactive state ( Supplementary Figure 1B), the homozygous 9bp deletion in KO1 suggests that the inactive X chromosome can be edited by the CRISPR/Cas9 system. Overall, out of 13 colonies that were sequenced, 11 contained INDELs thus giving a targeting efficiency of 85%.

To determine the genotype of the actively expressed *MECP2* mRNA in these cell lines, cDNA sequencing was performed ( Figure 2E). The 14bp deletion allele in KO2 appears to reside on the active X chromosome as all cDNA sequence reads from this cell line contained this out-of-frame deletion, highly indicative of a protein KO phenotype. Surprisingly the 9bp in-frame deletion in the middle of exon 3 of KO1 resulted in the whole of exon 3 being removed from the mature mRNA transcript, causing exons 2 and 4 to be spliced together in-frame. Western blot analysis confirmed the complete absence of any full length MeCP2 protein in both cell lines ( Figure 2G). In order to identify clones that might contain truncated protein, Western blot analysis was performed using two different antibodies, one against the N-terminus of MeCP2 and another against the C-terminus, and this revealed that KO1 has very low levels of a truncated protein ( Supplementary Figure 2B). Even though this cell line cannot technically be referred to as a protein KO cell line, the extremely low MeCP2 protein level that remains (and the removal of critical residues via deletion of exon 3) probably results in a cell line that is phenotypically null, as has been observed in mice ( Chen *et al.*, 2001). Finally, the top off-target loci for each sgRNA were sequenced for off-target INDEL formation and as expected based on recent findings in hiPSCs ( Paquet *et al.*, 2016), no off-target cutting was observed ( Figure 2H). These experiments confirm Cas9-induced INDEL formation to be successful, specific and highly efficient in LUHMES cells.

### Insertion of Rett syndrome-causing point mutations into *MECP2*

We also explored the possibility of introducing specific point mutations into LUHMES cells, historically a more challenging procedure for somatic cells ( Hendrie & Russell, 2005). The *MECP2* locus is an ideal candidate for use in optimising CRISPR knock-in (KI) conditions as there are a number of disease-causing point mutations throughout the locus ( Lyst & Bird, 2015). Furthermore the manipulation of this X-linked gene offers the opportunity to explore the ability of the CRISPR/Cas9 system to genetically manipulate genes on the inactive X chromosome.

In the previous experiment serial dilution was used to generate single cell colonies but we found that this method led to low efficiency of cloning and some colonies were derived from more than one genetically modified cell line, as several expressed alleles were detected in cDNA sequencing ( Supplementary Figure 2C). In order to improve clonal selection we used FACS sorting to cleanly isolate single cells into a 96-well plate. LUHMES cells were amenable to this manipulation, with approximately 50–60% of wells repopulating to produce single-cell colonies.

**Figure 3.**
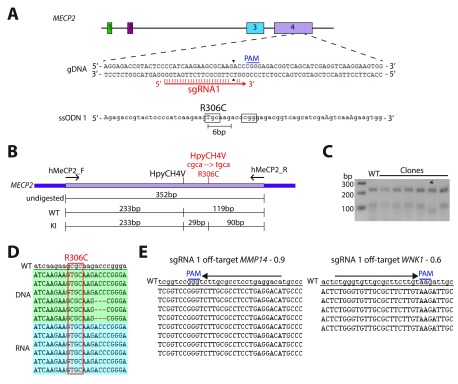
Generation of a human neuronal cell line containing a Rett syndrome-causing missense mutation in *MECP2*. ( **A**) Schematic representation of the *MECP2* locus with the sgRNA 1 target sequence labelled and the ssODN 1 donor molecule with point mutation alterations indicated in upper case. The site of double strand break is labelled with two arrowheads and the distance between the point mutation of interest and the double-strand break site is indicated. ( **B**) Schematic representation of the RFLP screening assay used for identifying positive knock-in clones. Mutation of arginine at position 306 to cysteine results in the introduction of a novel target sequence for the restriction enzyme HpyCH4V. Primers used for PCR amplification are labelled. ( **C**) HpyCH4V digests of the PCR product ( Supplementary Figure 3A) to identify clones that have gained a novel HpyCH4V target sequence. A positive clone is identified with an asterisk. ( **D**) Sequencing of genomic DNA and cDNA from the RFLP-positive cell line confirms the cell line to be *MECP2-R306C*. ( **E**) Sequencing of the top two off-target sites for sgRNA 1 in the R306C cell line. Number next to the locus name is the off-target score as calculated by crispr.mit.edu.

**Figure 4.**
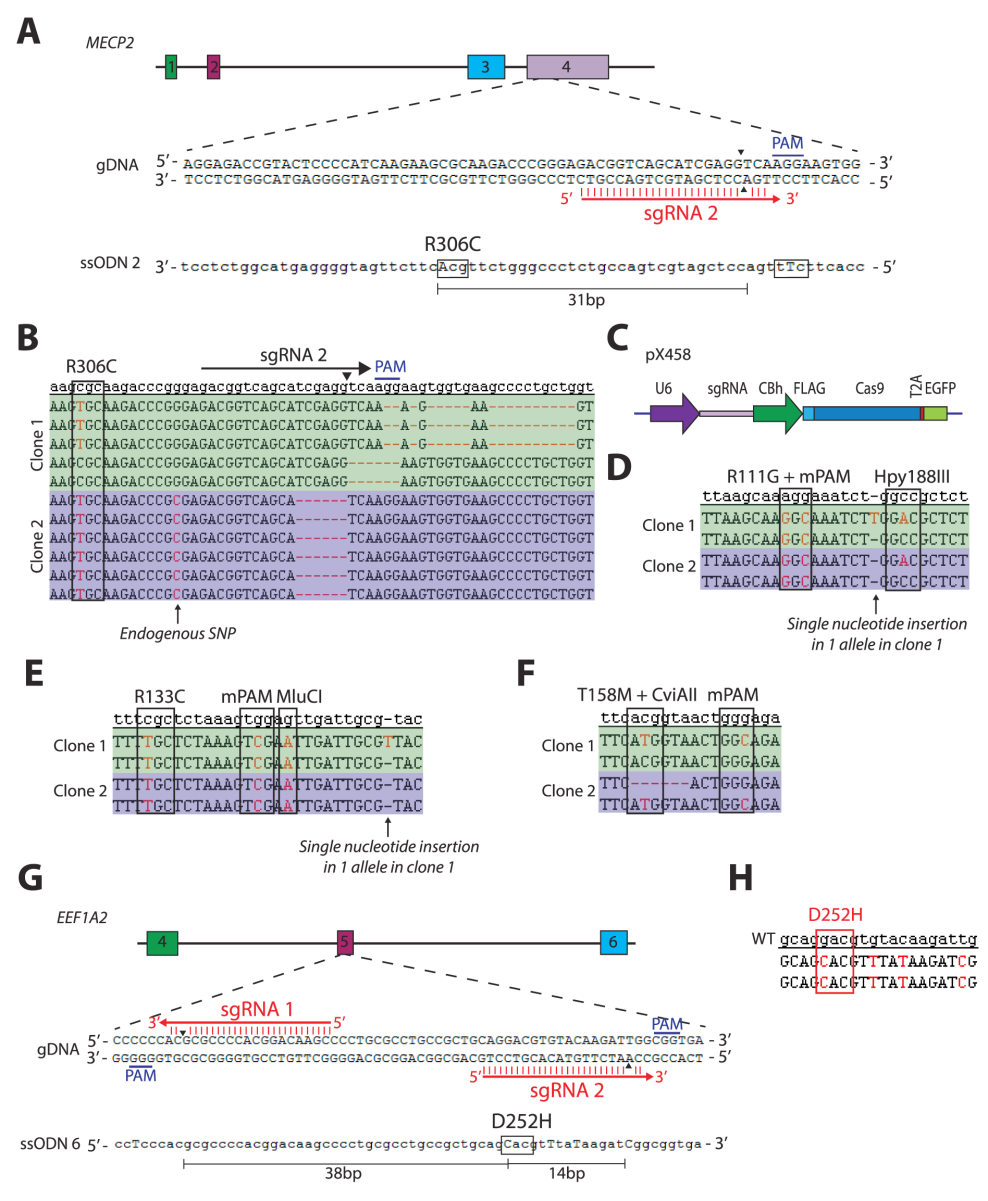
Generation of human neuronal cell lines containing missense mutations that cause neurological disorders. ( **A**) Schematic representation of the *MECP2* locus with the sgRNA 2 target sequence labelled. Site of double-strand break is indicated with arrowheads. ssODN molecule is shown with point mutation changes highlighted in upper case and the DSB to point mutation distance labelled. ( **B**) Genomic DNA sequencing of two R306C positive clones. Both clones have the correctly inserted R306C point mutation as well as downstream deletions induced by sgRNA 2. The site of DSB is indicated by an arrowhead. ( **C**) Schematic representation of a plasmid containing Cas9, sgRNA and EGFP expression constructs. ( **D**) Genomic DNA sequencing of two R111G positive cell lines. ( **E**) Genomic DNA sequencing of two R133C positive cell lines. ( **F**) Genomic DNA sequencing of two T158M positive cell lines. ( **G**) Schematic representation of part of the *EEF1A2* locus, with sgRNA target sequences labelled and the ssODN donor molecule with point mutation alterations indicated in upper case. Sites of single-strand nicks are indicated with an arrowhead. Distances between each nick and the point mutation of interest are labelled. ( **H**) Genomic DNA sequencing of the *EEF1A2-D252H* positive cell line.

First, the ability to knock-in the Rett syndrome-causing missense mutation of arginine at position 306 to cysteine (R306C) was tested ( Figure 3A). The R306C mutation itself (CGC➔TGC) creates a novel target sequence for the restriction enzyme HpyCH4V ( Figure 3B). This allowed for easy screening of genomic DNA from single-cell clones using a restriction fragment length polymorphism assay (RFLP), with the positive clones from this assay being confirmed by sequencing. Initially, a plasmid targeting vector containing 2044bp of homology was used to deliver the R306C point mutation and a silent PAM-abolishing mutation to prevent re-cutting of a recombined allele. Out of 191 single cell clones that were screened using the HpyCH4V RFLP assay, 0 appeared to be positive (experiment 1 in Table 1). Next a 110bp single-stranded oligodeoxynucleotide (ssODN) was used in combination with a non-complementary sgRNA that cuts 6bp away from R306, and in this instance 1 cell line out of 69 was positive, giving a KI efficiency of 1.6% ( Figure 3A+C+D, experiment 2 in Table 1). Again analysis of this cell line demonstrated no off-target cutting ( Figure 3E). An alternative sgRNA:ssODN pair where sgRNA 2 cuts 31bp away from R306, but the sgRNA and ssODN were complementary to one another was also tested ( Figure 4A). This combination produced two positive R306C cell lines (KI efficiency of 2.9%, experiment 3 in Table 1), but both cell lines contained downstream deletions at the site of the double-strand break (DSB) ( Figure 4B). These results suggest that a large distance between the point mutation and the DSB could be more susceptible to error-prone recombination and therefore subsequent INDEL formation.

**Table 1.** Point mutation KI efficiencies in the *MECP2* locus. PM = point mutation. DSB = double-strand break.

	Point mutation	Selection, plasmid amount	Donor molecule (ssODN is 10 μM)	sgRNA: ssODN	PM ➔ DSB distance	Number of upstream mutations	Number of KI clones	KI efficiency
1	R306C	Puro, 1.2 μg	1.9 μg 2 kb plasmid	-	31 bp	1	0/191	0%
2	R306C	Puro, 2 μg	4 μl 110 bp ssODN 1	Not comp	5 bp	1	1/69	1.6%
3	R306C	GFP, 2.5 μg	8 μl 110 bp ssODN 2	Comp	31 bp	1	2/69	2.9%
4	R111G	GFP, 2 μg	10 μl 100 bp ssODN 3	Comp	3 bp	0	7/27	26%
5	R133C	GFP, 2 μg	10 μl 100 bp ssODN 4	Comp	6 bp	2	2/54	3.7%
6	T158M	GFP, 2 μg	10 μl 100 bp ssODN 5	Comp	4 bp	1	1/18	5.5%
7	T158M	GFP, 2 μg	10 μl 100 bp ssODN 5	Comp	5 bp	1	1/13	7.7%

In an attempt to increase the efficiency of KI the CRISPR plasmid that encodes puromycin resistance was exchanged for a CRISPR plasmid that encodes for green fluorescent protein ( Ran *et al.*, 2013b) ( Figure 4C). Thus, instead of subjecting the Nucleofected LUHMES cells to puromycin selection and FACS sorting, these two steps were combined into one by using the presence of GFP in cells two days after Nucleofection to identify positively transfected cells and to sort them into a 96-well plate. Using this new strategy three new Rett syndrome-causing point mutations, R111G, R133C and T158M, were targeted using ssODNs ( Supplementary Figure 3). Two of these three point mutations do not introduce a novel restriction enzyme target sequence and so one was engineered into the ssODN for ease of screening. Each 100bp ssODN contained point mutations to introduce the following motifs: the mutation of interest, a silent PAM abolishing mutation, and a silent mutation to insert a novel restriction enzyme target sequence ( Supplementary Figure 3). All three targeting experiments generated positive cell lines as judged by genomic DNA sequencing ( Figure 4D,E,F) and as can be seen in Table 1, the efficiency of KI for all three point mutations is significantly increased relative to the initial efficiency of 1.6%, reaching a maximum of 26% for KI of R111G.

As shown in Table 1, several factors could contribute to the large variability in targeting efficiency. Firstly, the distance of the sgRNA-induced DSB from the point mutation of interest varies, and secondly the number of mismatched residues in the ssODN that are upstream of the DSB also varies. This latter variable would be in line with evidence that mismatches in the non-sgRNA binding DNA strand upstream of the PAM are refractory to homology-directed repair (HDR) ( Richardson *et al.*, 2016). Despite uncertainty regarding the exact constraints on efficient KI of point mutations using CRISPR technology, KI efficiencies in the somatic LUHMES neuronal progenitor cells are sufficient to allow the rapid generation of cell lines containing disease-causing point mutations with minimal clonal selection and screening.

### Insertion of a neurodevelopmental disorder-causing point mutation into *EEF1A2*

To demonstrate the utility of LUHMES cells as a model system for other neurological disorders, and to confirm efficient KI at an autosomal locus, we targeted the D252H missense mutation in the *EEF1A2* gene that causes severe neurodevelopmental delay and intellectual disability ( Nakajima *et al.*, 2015). The approach was to use two sgRNAs that cut 51bp apart, combined with Cas9 nickase protein ( Ran *et al.*, 2013a) ( Figure 4G) and a 200bp ssODN. Again the presence of GFP expression in LUHMES cells was used to identify positively transfected cells and to sort single cells into 96-well plates. In this experiment a KI efficiency of 14% was achieved and interestingly the D252H positive cell line has a KI on both alleles and is therefore a homozygous, true positive cell line ( Figure 4H). The successful knock-in of a point mutation into an autosomal locus and subsequent generation of an *EEF1A2-D252H* cell line demonstrates the ease of genetic manipulation of LUHMES cells and highlights its utility for modelling a variety of human neurogenetic disorders.

### Knock-in of a large tag into the endogenous *MECP2* locus

**Figure 5.**
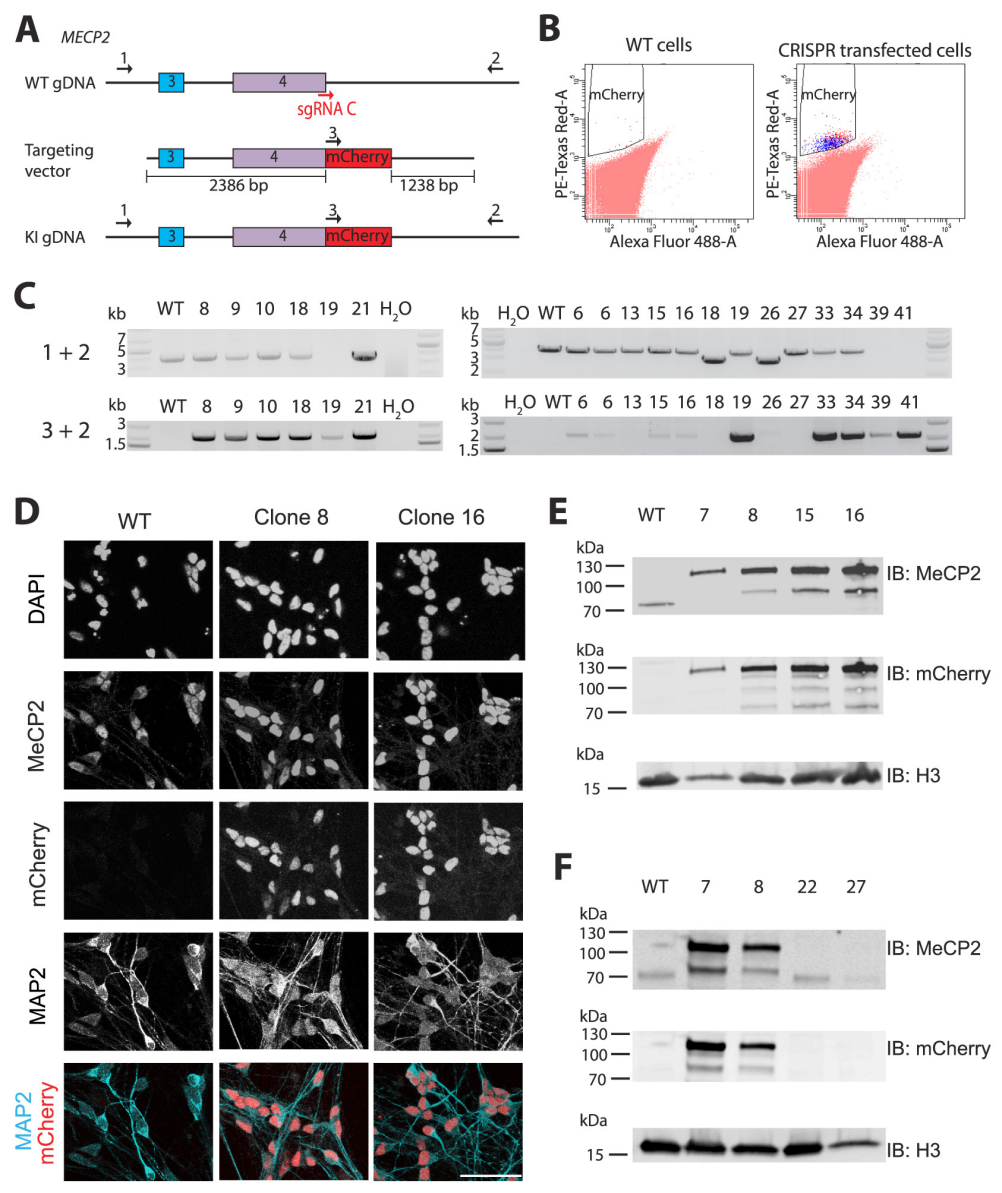
Endogenous knock-in of an mCherry tag into the *MECP2* locus in LUHMES cells. ( **A**) Schematic representation of the *MECP2* locus with the sgRNA C target region labelled, the targeting vector, and the recombined genomic DNA allele. Positions of primers used for screening purposes in ( **C**) are indicated. ( **B**) Flow cytometry analysis of WT cells and cells that were transfected with Cas9, sgRNA and targeting plasmid. ( **C**) PCR screening of genomic DNA from single-cell clones that were identified as being mCherry positive by flow cytometry. ( **D**) Immunofluorescence imaging of WT cells and two single-cell clones using DAPI and antibodies probing for MeCP2 and MAP2. Images are slices through a z-stack. Scale bar represents 50 μm. ( **E**) Immunoblot analysis of WT cells and four *MECP2-mCherry* positive clones. ( **F**) Immunoblot analysis of WT cells, two *MECP2-mCherry* positive clones and two *MECP2-mCherry* negative clones. ( **E** **+** **F**) Top panel probe: MeCP2. Middle panel probe: mCherry. Bottom panel probe: Histone H3 as a loading control.

Finally, we assessed the ability of the CRISPR/Cas9 system to introduce a large tag into an endogenous locus in LUHMES cells. Again the *MECP2* locus was targeted and a sgRNA that spans the stop codon was used, resulting in its targeting sequence being abolished once a successful KI has occurred ( Figure 5A). We chose to KI mCherry and use FACS analysis to provide an accurate estimate of KI efficiency, i.e. the percentage of mCherry positive cells in the whole population. Due to the large size of mCherry (711bp), a plasmid donor was used for targeting with 2.3kb and 1.2kb homology arms ( Figure 5A). FACS analysis determined the percentage of mCherry positive cells in the entire population to be 0.015% ( Figure 5B). Out of 29 single-cell clones assessed, 25 were *MECP2-mCherry* positive as judged by a PCR assay that used a forward primer in mCherry itself and a reverse primer in the *MECP2* gene locus, outwith the targeting vector ( Figure 5C). Positive cell lines were confirmed by immunofluorescence and Western blot analysis ( Figure 5D+E; Supplementary Figure 4), and two negative cell lines (as determined by PCR analysis) were confirmed by Western blot ( Figure 5F, clones 22 + 27). These experiments demonstrate successful CRISPR-mediated KI of a large tag into LUHMES cells, thus highlighting the variety of genetic alterations that are feasible in this cell line.

## Discussion

The LUHMES cell line is an immortalised neuronal cell line derived from an 8-week old female foetus that is highly proliferative in a stem-cell like, yet neuronal-committed state, and can differentiate into mature dopaminergic neurons via addition of tetracycline to the cell culture medium ( Scholz *et al.*, 2011). A key advantage of LUHMES cells compared to other neuronal differentiation systems is the near 100% homogeneity of differentiation into a population of mature, post-mitotic neurons, without the presence of astrocytes or other non-neuronal cell types. This homogeneity is extremely beneficial for “bulk population” experiments such as RNA-sequencing, Western blot analysis and Hi-C studies where mixed cell populations could result in skewed data and difficult-to-interpret results. Here we describe methods for the successful genetic manipulation of LUHMES cells in order to create targeted protein knock-out, disease-causing point mutation knock-in, and large tag knock-in cell lines.

The combination of targeted mutagenesis with rapid generation of mutant neuronal cells provides a potentially valuable tool for neuroscience. These manipulated cell lines may complement *in vivo* datasets as disease phenotypes obtained using mouse models can be coupled with electrophysiological and biochemical data from human neurons in order to bridge the gaps between disease causing mutations, neuronal malfunction and whole organism pathophysiology. The ease of high-throughput differentiation of this cell line in 96-well plates opens the door towards drug screening programs like those already being pursued using hiPSCs ( Cao *et al.*, 2016; Lee *et al.*, 2012). As a potential alternative, LUHMES cells simplify the differentiation procedure, speed up the differentiation time course, and ensure that a homogeneous population of mature neurons will be screened. Furthermore, although downstream applications of genetically modified LUHMES cells might be limited by their dopaminergic lineage, removal of supplements from the differentiation medium results in the production of tyrosine hydroxylase-negative cells, that differentiate into morphologically and immunocytologically mature neurons for study ( Scholz *et al.*, 2011).

### Bi-allelic X chromosome targeting

**Figure 6.**
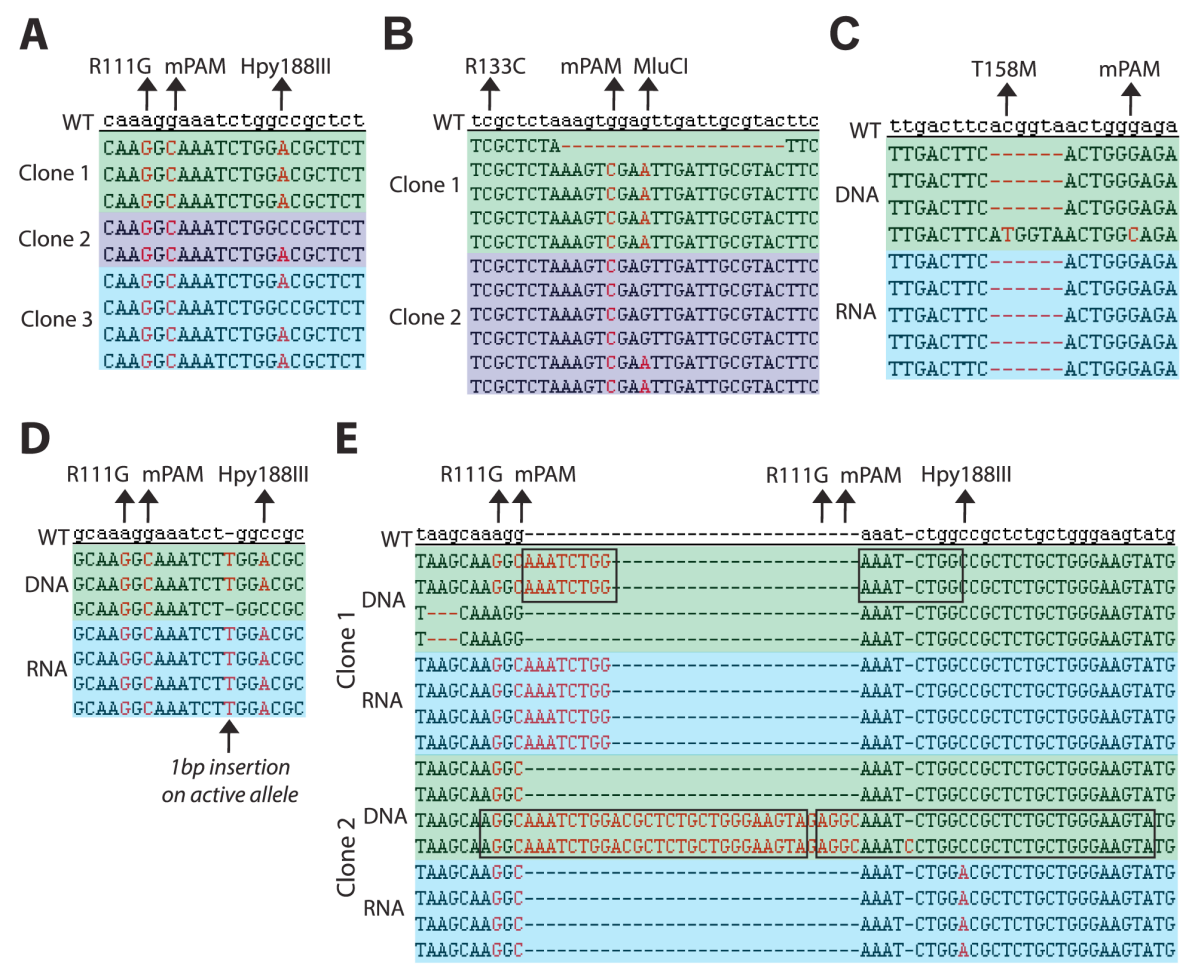
Analysis of range of mutations induced by CRISPR/Cas9 in LUHMES cells. ( **A**+ **B**) Partial recombination can occur with the ssODN molecule. ( **A**) Three single cell clones from the R111G targeting experiment, two of which contain a partially recombined allele. ( **B**) Two single cell clones from the R133C targeting experiment; clone 1 has an allele with 2/3 of the KI residues, clone 2 has an allele with only 1/3 of the KI residues. ( **C**) Genomic DNA sequencing of a single clone reveals one allele to contain two point mutations and the other allele to contain a 6bp deletion, yet cDNA sequencing identifies the HDR to have occurred with the allele on the inactive X chromosome. ( **D**) cDNA sequencing identifies the allele on the active X chromosome having undergone recombination, with all three point mutations integrated plus a single base pair insertion. ( **E**) Sequencing of single-cell clones reveals duplication events occurring as a result of multiple recombination events with the ssODN. ( **A**, **B**, **C**, **D**, **E**) mPAM - silent point mutation that abolishes the PAM site for each sgRNA. Rett-syndrome causing point mutations of interest and silent restriction enzyme target sequence point mutations are highlighted with arrows.

We observed that both alleles undergo HDR at a rather high frequency, regardless of whether the 2 ^nd^ allele is on an autosome or the inactive X chromosome. For the *MECP2-R111G* targeting experiment, out of the seven cell lines that contained a KI of R111G on the active X allele, six also contained a KI on the inactive X allele. Likewise for the *MECP2-R133C* targeting experiment, the two R133C positive cell lines had undergone HDR repair on both alleles. It is however important to note that not all HDR events result in a clean integration. Partial recombination within the short distance of a 100bp ssODN ( Figure 6A+B) and multiple integrations of the ssODN in tandem at a locus have been observed ( Figure 6E), as well as recombined alleles containing INDELs at the site of sgRNA DSB ( Figure 4B).

Surprisingly we found in more than one case that the *MEPC2* allele on the inactive X chromosome underwent HDR, while the allele on the active X chromosome acquired an INDEL. It is expected that the active allele would be more open and accessible to recombination compared to the inactive X chromosome, however at least two cell lines were observed that have an active allele INDEL and an inactive allele KI ( Figure 6C+D). These data reflect the large variety of genomic alterations that can be induced by CRISPR/Cas9 and demonstrate the somewhat unpredictable nature of HDR-mediated gene targeting. Our experiments stress the need for a sgRNA-induced DSB as close as possible to the desired genetic alteration and highlight the importance of donor molecule design; in particular use of a sgRNA-complementary ssODN that has minimal mismatches upstream of the PAM seems to be most efficient.

### Further optimisation of KI of large tags

In the mCherry KI experiment the plentiCRISPR plasmid ( Sanjana *et al.*, 2014) was used to deliver Cas9 and sgRNA, and positively transfected cells were selected using the co-encoded puromycin resistance gene ( Figure 2B), while the mCherry targeting vector was delivered as a separate plasmid. It is possible that a proportion of transfected cells did not take up both plasmids ( Assur *et al.*, 2012) and this could explain the low targeting efficiency of 0.015% observed in this experiment. As such, a double antibiotic selection method could increase HDR efficiencies when plasmid donors are necessary, for example by including an expression cassette for the bacterial blasticidin resistance gene (bsr) in the targeting plasmid and selecting with both puromycin and blasticidin.

Design of targeting vectors with alternative homology arm lengths could also improve the efficiency of KI. Indeed, others have reported comparable efficiencies with homology arms of 175 bp compared to 700–900 bp in human cell lines ( Natsume *et al.*, 2016). Alternatively, the use of drugs to inhibit the NHEJ pathway could also boost HDR in LUHMES cells ( Maruyama *et al.*, 2015; van Overbeek *et al.*, 2016). Even without these enhancements, the power of FACS sorting allows efficient selection for the small number of positive cells within a large population and, as demonstrated here, this results in a stream-lined and efficient protocol for CRISPR-mediated tag KI in LUHMES cells.

### Concluding remarks

**Figure 7.**
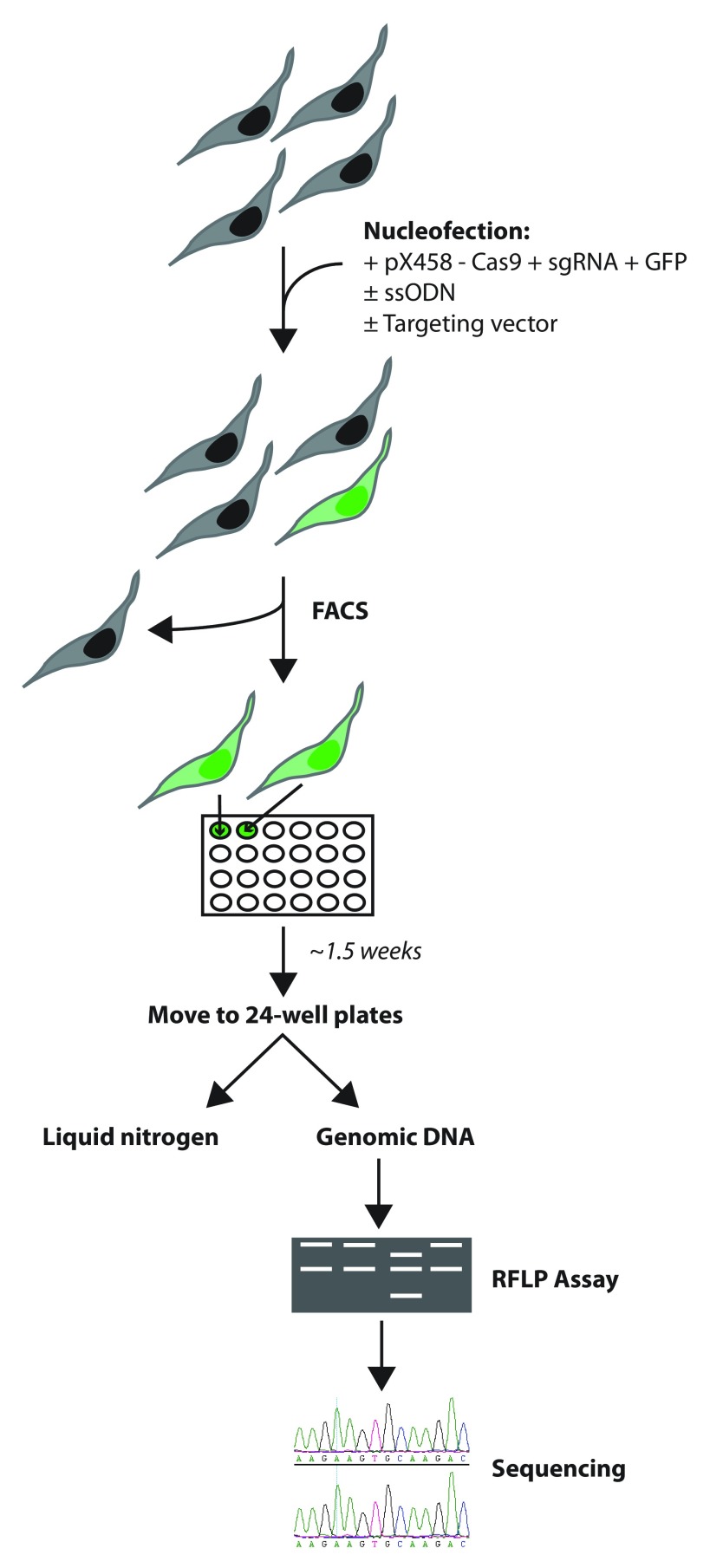
Simple targeting pipeline for generation of genetically modified LUHMES cell lines using CRISPR technology. Cells are Nucleofected with the plasmids and ssODNs necessary for the specific targeting experiment and taken for FACS sorting two days later. After approximately 10 days single cell colonies will have expanded enough to require dissociation and transfer to 24-well plates. From 24-well plates cells can be frozen down for storage in liquid nitrogen and some cells harvested for genomic DNA. The first step of the screening strategy is to perform an RFLP assay to identify a subset of clones that will be taken for genomic DNA sequencing which will identify cell lines that are positive for a clean KI. For more detailed information see methods and materials.

In conclusion, we have demonstrated efficient genetic manipulation of the LUHMES female human neuronal cell line to create a number of lines harbouring neurological disease-causing point mutations ( Figure 7). The future phenotypic assessment of these cell lines will provide significant insight into the molecular mechanisms of these diseases. Using the methods described here LUHMES cells have the potential to be a valuable tool for exploration of the underlying biology of neurogenetic disorders and may pave the way for drug development and therapeutic strategies in the future.

## Experimental procedures

### Plasmids and cloning

All CRISPR plasmids were purchased from Addgene and originated from the Zhang lab; pX458 (48138), pX461 (48140) and plentiCRISPRv2 (52961). The crispr.mit.edu online webtool was used for sgRNA design ( Table 2). Cloning of sgRNAs into CRISPR plasmids was performed following the protocol from the Zhang lab, available online at genome-engineering.org and described in Ref ( Cong *et al.*, 2013). All ssODNs were ordered as 100bp or 110bp (Sigma, desalted) or 200bp (IDT, PAGE-purified) oligonucleotides ( Table 3). Gateway cloning (Invitrogen) was used to create the mCherry targeting plasmid. The pmaxGFP plasmid for optimisation of Nucleofection techniques came in the Nucleofection kit provided by Lonza.

**Table 2.** Sequences of sgRNAs used in this study. All sgRNAs are 20 nucleotides long. Those without a 5' G nucleotide had one added for efficient U6 promoter transcription to make a 21 nucleotide sequence.

sgRNA name	Experiment	Sequence (5’ ➔ 3’)
sgRNA A	Knock-out of MeCP2	AGAAGCTTCCGGCACAGCCG
sgRNA B	Knock-out of MeCP2	CGCTCCATCATCCGTGACCG
sgRNA 1	Knock-in of *R306C-MECP2*	CCATCAAGAAGCGCAAGACC
sgRNA 2	Knock-in of *R306C-MECP2*	GACGGTCAGCATCGAGGTCA
sgRNA 3	Knock-in of *R111G-MECP2*	GGACACGGAAGCTTAAGCAA
sgRNA 4	Knock-in of *R133C-MECP2*	AAAAGCCTTTCGCTCTAAAG
sgRNA 5	Knock-in of *T158M-MECP2*	GATTTTGACTTCACGGTAAC
sgRNA 6	Knock-in of *T158M-MECP2*	ATTTTGACTTCACGGTAACT
sgRNA 1	Knock-in of *D252H-EEF1A2*	GCTTGTCCGTGGGGCGCGTG
sgRNA 2	Knock-in of *D252H-EEF1A2*	CAGGACGTGTACAAGATTGG
sgRNA C	Knock in of mCherry tag into *MECP2*	TTAGCTGACTTTACACGGAG

**Table 3.** Sequences of ssODNs used in this study. Point mutation changes from the wild-type, endogenous sequence are highlighted in bold, with the point mutation of interest in bold red.

ssODN	Experiment	Sequence (5’ ➔ 3’)
1	Knock-in of *R306C-MECP2*	GAGTCTTCTAT **T**CGATCTGTGCA **A**GAGACCGTACTCCCCATCAAGAAG **T**GCAAGACCCGGGAGACGGT CAGCATCGA **A**GTCAA **A**GAAGTGGTGAAGCCCCTGCT **A**GTGTC
2	Knock-in of *R306C-MECP2*	GACACCAGCAGGGGCTTCACCACTTCCTTGACCTCGATGCTGACCGTCTC **G**CGGGTCTTGC **A**CTTCT TGATGGGGAGTACGGTCTCCTGCACAGATCG **A**ATAGAAGACTC
3	Knock-in of *R111G-MECP2*	CTTACTTACTTGATCAAATACACATCATACTTCCCAGCAGAGCG **T**CCAGATTT **G**C **C**TTGCTTAAGCTTCCGT GTCCAGCCTTCAGGCAGGGTGGGGTCAT
4	Knock-in of *R133C-MECP2*	CAGGGATGTGTCGCCTACCTTTTCGAAGTACGCAATCAA **T**TC **G**ACTTTAGAGC **A**AAATGCTTTTCCCTGGG GACTGTGGGGACAAACAGAAAGACACAAG
5	Knock-in of *T158M-MECP2*	GGCTTCTTAGGTGGTTTCTGCTCTCGCCGGGAGGGGCTCCCTCT **G**CCAGTTACC **A**TGAAGTCAAAAT CATTAGGGTCCAGGGATGTGTCGCCTACCTTTT
6	Knock-in of *D252H-EEF1A2*	TAAGGAGGGCAACGCAAGCGGCGTGTCCCTGCTGGAGGCCCTGGACACCATCCTGCC **T**CCCACG CGCCCCACGGACAAGCCCCTGCGCCTGCCGCTGCAG **C**ACGT **T**TA **T**AAGAT **C**GGCGGTGAGCAAGGGC GCTGTGCTGGAGCTCCTGCCTGGCCAGCTCTGCCTGCCCTAGACCAGGGGCCCCTACAAGGCATCTCAA

The R306C targeting plasmid was created by firstly performing a PCR reaction using Phusion polymerase in GC buffer (NEB) with approximately 100–250 ng LUHMES genomic DNA in a 25 μl reaction volume ( Table 4). Subcloning was performed using 2 μl of this PCR mix with the Zero Blunt TOPO PCR cloning kit (Invitrogen) and 2 μl of the subcloning reaction was transformed into DH5α cells. The following day two colonies were picked into 3 ml cultures of LB/kanamycin and grown overnight by shaking at 37°C. The next day plasmids were extracted from 1.5 ml of culture using the Plasmid Miniprep kit (Qiagen) and sequenced using primers spanning the *MECP2* locus. After sequencing confirmed error-free incorporation of the *MECP2* fragment into the pTOPO vector, 50 ml cultures of LB/kanamycin were set up using the remaining 1.5 ml of bacterial culture and grown overnight by shaking at 37°C. The next day plasmids were extracted using the Plasmid Maxiprep kit (Qiagen). This plasmid was subjected to site-directed mutagenesis using the QuickChange II XL kit (Agilent) following the manufacturer’s instructions. Two rounds of mutagenesis PCRs were used to incorporate first the R306C point mutation, and then two PAM-abolishing point mutations for two separate sgRNAs to create the final targeting vector.

**Table 4.** Sequences of primers used in this study. For Gateway cloning primers, sequence in red is the att tag, sequence in black is homologous to *MECP2* or mCherry.

Experiment	Primer name	Sequence (5’ ➔ 3’)
Sequence CRISPR plasmids	pLKO1_5	GACTATCATATGCTTACCGT
Cloning of pR306C targeting vector	hMeCP2_6F	CGCTCTGCTGGGAAGTATGA
	hMeCP2_5R	CCAACTACTCCCACCCTGAA
Site-directed mutagenesis of pR306C targeting vector	R306C_F	TCCCCATCAAGAAGTGCAAGACCCGGGAG
	R306C_R	CTCCCGGGTCTTGCACTTCTTGATGGGGA
	PAMs_F	ATCGAGGTCAAAGAAGTGGTGAAGCCCCTGCTAGTGTCCACCCTCG
	PAMs_R	CGAGGGTGGACACTAGCAGGGGCTTCACCACTTCTTTGACCTCGAT
Gateway cloning of mCherry targeting vector	attB1_MeCP2_intron2F	GGGGACAAGTTTGTACAAAAAAGCAGGCTCCACAGCCCAAATTCCTAAA
	attB4_MeCP2_stopR	GGGGACAACTTTGTATAGAAAAGTTGGGTG GCTAACTCTCTCGGTCACGG
	attB4r_mCherry_IF_Tm	GGGGACAACTTTTCTATACAAAGTTGTTATGGTGAGCAAGGGCGA
	attB3r_mCherry_IF_Tm	GGGGACAACTTTATTATACAAAGTTGTCTACTTGTACAGCTCGTCCATGC
	attB3_MeCP2_stopF	GGGGACAACTTTGTATAATAAAGTTGTGACTTTACACGGAGCGGAT
	attB2_MeCP2_8R	GGGGACCACTTTGTACAAGAAAGCTGGGTAGCAGAAATGGAAGGGGAGAA
Phusion PCR for R306C clones	hMeCP2_F	AGCTCCTTGTCAAGATGCCT
	hMeCP2_R	AGTCCTTTCCCGCTCTTCTC
Phusion PCR for R111G clones	Intron2_2F	TCCCTTGAAGTGCGACTCAT
	1_R	CCTCTCCCAGTTACCGTGAA
Phusion PCR for R133C clones	Intron3_2F	CAGACGAGTGAGTGGCTTTG
	2_R	AGTCCTTTCCCGCTCTTCTC
Phusion PCR for T158M clones	Intron3_2F	CAGACGAGTGAGTGGCTTTG
	3_R	CAATCCGCTCCGTGTAAAGT
Phusion PCR for *MECP2-* *mCherry* clones	1	TCCCTTGAAGTGCGACTCAT
	2	GGACGGAGGAAGGGAAAGAA
	3	GGGGACAACTTTTCTATACAAAGTTGTTATGGTGAGCAAGGGCGA
Phusion PCR for *EEF1A2-* *D252H* clones	hD252H_1F	TTCCTCATCTCAAAGGGCACG
	hD252H_2R	CAAGTTTAGCCTGAACAGCAGTA
Sequencing of *EEF1A2-* *D252H* clones	hD252H_2F	CCCACAGAAGTGTGTGGTAAG
	hD252H_2R	TTGGAGACAGCCAGTCTTG

### Tissue culture

LUHMES cell (ATCC cat# CRL-2927, RRID CVCL_B057, kind gift from Dr Tanja Waldmann) tissue culture medium and methods were as described in Reference ( Scholz *et al.*, 2011) with some minor alterations. All vessels were coated in poly-L-ornithine (PLO) and fibronectin overnight at 37°C. Proliferating LUHMES cells were seeded at 2x10 ^6^ cells/T75 every 2 days. For differentiation, 2.5x10 ^6^ cells were seeded in a T75 for the first 2 days of the protocol and on day 2 cells were seeded as follows: 8x10 ^6^ cells/T75 and 0.15x10 ^6^ cells/coverslip. During differentiation a half-media change was performed on day 6 and neurons were harvested for protein or fixed for immunostaining on day 9.

### Transfection methods

LUHMES cells were transfected by Nucleofection (Lonza) using a Basic Nucleofector kit for primary neurons (VAPI-1003) and a Nucleofector II device. LUHMES cells were dissociated with 4ml of trypsin (Gibco), centrifuged at 13000 rpm for 5 minutes and resuspended in PBS for cell counting using a Scepter device (Millipore). Aliquots of 2x10 ^6^ cells were pipetted into 15 ml falcons and these were centrifuged for 5 minutes at 13000 rpm. PBS was removed from all 15 ml falcons and the appropriate volume of each plasmid/ssODN was added to each tube (see Table 1). One by one cells and plasmids were then resuspended in 90 μl of the Nucleofection solution and immediately transferred into a cuvette (provided in the kit) for electroporation in the Nucleofector II device. After electroporation, RPMI medium (Sigma) was added using the pipette provided in the kit and cells were moved into 15 ml falcons and incubated at 37°C for 5 minutes before plating out into 6-well plates containing pre-warmed LUHMES proliferation media. Media was changed after a minimum of 4 hours.

### FACS sorting

For flow cytometry sorting of single LUHMES cells into each well of a 96-well plate, Greiner plates were pre-coated overnight in PLO/fibronectin as described in Reference ( Scholz *et al.*, 2011). Proliferation medium was supplemented with 1X B27 (Sigma), 100 U/ml penicillin (Gibco) and 100 μg/ml streptomycin (Gibco) and 100 μl was added to each well of the 96-well plates. LUHMES cells were trypsinised and centrifuged in a 15 ml falcon tube at 13000 rpm for 5 minutes and resuspended in 1 ml of Advanced DMEM/F12 supplemented with 10 μM HEPES. Cells were sorted using a 100 μm nozzle with a FACSaria machine at room temperature. Six days later 100 μl of proliferation medium was added to the 96-well plates.

### Targeting pipeline in LUHMES cells

Day 1 – Thaw low passage number LUHMES

Day 3 – Passage once

Day 5 – Nucleofect and change media after 4 hours

Day 7 – Take for FACS sorting of single GFP-positive cells into a 96-well plate

Day 13 – Top up 96-well plate with 100 μl of media

Day 16 – Start moving clones from 96-well plates to 24-well plates

Day 17 onwards – Split individual clones in 24-well plates as they are confluent, freeze down half the well for liquid nitrogen, move the other half to a new well in a 24-well plate for genomic DNA.

### Genomic DNA isolation

For extracting genomic DNA from tissue culture samples, Puregene Core Kit A (Qiagen) was used following the manufacturer’s instructions. Occasionally, when RNA and DNA were required from a single sample, the Allprep DNA/RNA Mini kit (Qiagen) was used.

For large scale genomic DNA extraction from LUHMES single cell clones in a 24-well plate an alternative approach was used. Individual clones were trypsinised and harvested when confluent and incubated in 400 μl lysis buffer (50 mM Tris pH 9.0, 20 mM EDTA pH 8.0, 40 mM NaCl, 1% SDS, 0.5 mg/ml proteinase K) overnight at 55°C. The next day 300 μl saturated NaCl was added to each sample, mixed by vigorous shaking for 1 minute, and centrifuged at 14000 rpm for 10 minutes at room temperature. The supernatant was transferred to an Eppendorf containing 500 μl isopropanol, mixed by inversion and centrifuged at 14000 rpm for 10 minutes at 4°C. The DNA pellet was washed with 750 μl 70% ethanol, mixed by inversion, and centrifuged at 14000 rpm for 10 minutes at 4°C. The DNA pellet was air dried, resuspended in 50 μl TE and allowed to dissolve at 55°C overnight.

### RNA isolation and preparation of cDNA

RNA was extracted using an RNeasy Mini Kit (Qiagen) following manufacturer’s instructions. RNA concentration was measured using a Nanodrop 1000 (Thermo Scientific) and 10 μg RNA was taken for DNase1 treatment (Ambion) for 1 hour at 37°C. DNA-free RNA concentration was measured using a Nanodrop and 1 μg used for cDNA synthesis using qScript cDNA Supermix (Quanta).

### T7 Endonuclease I assay

Phusion polymerase in GC buffer (NEB) was used to PCR amplify 100–250 ng genomic DNA in a 50 μl reaction volume ( Table 4) and 5 μl of the reaction was ran out on an agarose/TAE gel. To the remaining 45 μl PCR mix, 5 μl of Buffer 2 (NEB) was added and the reactions were heated at 95°C for 10 minutes in a PCR machine and cooled slowly to 25°C to produce heteroduplexes. Each reaction was split in half and to one half 1 μl of T7E1 (NEB) was added. All reactions were incubated at 37°C for 30 minutes, followed by addition of 10 μl of 6X loading dye and analysis on an agarose/TAE gel.

### Restriction fragment length polymorphism assay

Phusion polymerase in GC buffer (NEB) was used to PCR amplify 100–250 ng genomic DNA in a 25 μl reaction volume ( Table 4) and 10 μl of each PCR was run out on an agarose/TAE gel. To the remaining PCR mix 29 μl of H _2_O, 5 μl of CutSmart buffer (NEB) and 0.5 μl of the appropriate enzyme was added. Reactions were incubated at the necessary temperature and length of time for each enzyme and were analysed by electrophoresis in an agarose/TAE gel.

### PCR and sequencing analysis

Phusion polymerase in GC buffer (NEB) was used to PCR amplify 100–250 ng genomic DNA in a 25 μl reaction volume ( Table 4) and 10 μl of the reaction was ran out on an agarose/TAE gel for confirmation of efficient PCR amplification. Each amplicon was subcloned using the Strataclone blunt PCR cloning kit (Agilent Technologies) following the manufacturer’s instructions and transformed into Strataclone Solopack competent bacteria (Agilent Technologies). The next day single colonies were picked and colony PCR was performed using either Phusion polymerase or DreamTaq polymerase (Thermo Fisher) in a 25 μl volume ( Table 4). For confirmation of PCR, 10 μl of each colony PCR reaction was analysed by agarose/TAE gel electrophoresis. Positive colony PCRs were treated with 0.25 μl Exonuclease I (NEB) and 0.25 μl FastAP alkaline phosphatase (Thermo) at 37°C for 15 minutes followed by inactivation at 85°C for 15 minutes. Sequencing was performed using BigDye Terminator v2.1 reaction mix with 3.5 μl of each sample and 3.2 pmol of primer.

### Western blot analysis

Whole cell protein extracts were prepared by homogenising cell pellets in NE1 buffer (10 mM HEPES pH 7.9, 10 mM KCl, 1 mM MgCl _2_, 0.5 mM DTT, 0.1% Triton-X 100, 20% glycerol and 1X protease inhibitor cocktail (Roche)). Homogenates were treated with benzonase for 15 minutes at room temperature and then measured for protein concentration using a Bradford assay (Protein Assay Dye Reagent concentrate, BioRad). Extracts were loaded onto pre-cast 4–20% Mini-PROTEAN TGX gels (BioRad) and ran at 200V for approximately 30–40 minutes. Gels were transferred onto nitrocelloluse membrane by transfer at 30V overnight at 4°C. Membranes were blocked in 5% milk, 0.1% Tween-20 in TBS for 30 minutes and probed with primary antibodies ( Table 5) in blocking solution for 1 hour at room temperature. IRDye 800CW α-mouse and IRDye 680LT α-rabbit secondary antibodies (Licor) were probed for 1 hour at room temperature in blocking buffer at a concentration of 1:10000 and scans were taken using a Licor Odyssey machine.

**Table 5.** Details of antibodies used in this study. Antibodies used for Western blot analysis (WB) or immunofluorescence (IF). Cat number – catalogue number.

Antibody	Company + Cat Number	Experiment	Dilution	Species	RRID
MeCP2	Sigma 7443	WB; IF	1000X	Mouse monoclonal	AB_477235
MeCP2	Sigma 6818	WB	1000X	Mouse monoclonal	AB_262075
GAPDH	Cell Signalling 5174	WB	5000X	Rabbit monoclonal	AB_10622025
H3	Abcam 1791	WB	5000X	Rabbit polyclonal	AB_302613
mCherry	Abcam 167453	WB, IF	1000X	Rabbit polyclonal	AB_2571870
Neurofilament	Covance SMI-311R-100	IF	500X	Mouse monoclonal	AB_509991
MAP2	Abcam 5392	IF	5000X	Chicken polyclonal	AB_2138153

### Immunofluorescence imaging

Cells grown on coverslips were fixed in 4% formaldehyde for 10 minutes, permeabilised with 0.2% Triton-X/PBS for 10 minutes and blocked for 30 minutes in 10% fetal bovine serum in PBS (FBS/PBS). Coverslips were incubated in the presence of primary antibodies ( Table 5) in 1% FBS/0.1% Tween-20/PBS for one hour room temperature. Coverslips were washed with 0.1% Tween-20/PBS, and incubated for one hour at room temperature with Alexa Fluor secondary antibodies (1000X dilution, Invitrogen) in 1% FBS/0.1% Tween-20/PBS. Secondary antibodies: α-mouse 488, α-rabbit 555 and α-chicken 633. Coverslips were finally stained with DAPI (5000X dilution in PBS) for 10 minutes at RT and mounted onto microscope slides using Prolong Diamond solution (Thermo Fisher). Z-stack images were taken using a Leica SP5 confocal microscope and z-stacks were flattened and processed using ImageJ 1.47v software.

### Other microscopy

Phase contrast images were taken on an Eclipse TS100 inverted microscope (Nikon) using QCapture Pro software version 5.1.1.14 (QImaging). UV fluorescence was used to take GFP pictures of cells. The IncuCyte microscope (Essen Biosciences) was used to take phase contrast and GFP fluorescent images and ZOOM software (Essen Biosciences) was used to extract and export the images.

### RNA FISH

RNA FISH was performed as described in ( Chaumeil *et al.*, 2008). BAC-based probes were used for detection of *MECP2* and *ATRX* (RP11-119A22 and RP11-42M11 respectively). Probes for *XIST* RNA detection have been used previously, as described in ( Chaligne *et al.*, 2015).

## Data availability

Zenodo: Efficient and versatile CRISPR engineering of human neurons in culture to model neurological disorders, DOI 10.5281/zenodo.163342 ( Shah *et al*., 2016)

## Supplementary Material

**Supplementary Figure 1. Optimisation of Nucleofection in LUHMES cells**.

( **A**) Karyotyping of LUHMES pre-neuronal cells confirms a normal ploidy. ( **B**) RNA FISH of these female cells demonstrates that X inactivation is already established in the pre-neuronal cells. Scale bar is 5 μm. ( **C**) Trials of various electroporation programs using the Basic Nucleofector kit for primary neurons. ( **D**) Comparison of different plasmid amounts on cell viability. ( **E**) Comparison of phenol:chloroform:isoamylalcohol plasmid purification on cell viability. ( **F**) Comparison of cell viability when endotoxin-free plasmids are used, when the cells are incubated at 37°C in RPMI for 5 minutes (RPMI) and when they are centrifuged before plating (spin). All scale bars are 200 μm.

**Supplementary Figure 2. Analysis of MeCP2 KO cell lines**.

( **A**) Representative phase contrast and GFP fluorescence images of LUHMES cells 25 hours after Nucleofection. Scale bar is 200 μm. ( **B**) Immunoblot detection of MeCP2 protein in single cell clones derived from the plentiCRISPR targeting experiment using an N-terminal and a C-terminal antibody. GAPDH is probed for a loading control. Note: E10 = WTC, D10 = KO1 and H4 = KO2 in Figure 2. ( **C**) Sequencing of cDNA from single LUHMES clones identifies mixed cell populations by the presence of multiple cDNA alleles per clone. Each colour block denotes an individual clone.

**Supplementary Figure 3. Optimisation of CRISPR-mediated point mutation knock-in of Rett syndrome causing missense mutations**.

( **A**) hMeCP2_F and hMeCP2_R PCR of WT cells and clones that have been targeted with components shown in Figure 3A, in preparation for RFLP analysis. ( **B, C, D**) Schematic representation of R111G (B), R133C (C) and T158M (D) targeting experiments. Two sgRNAs are labelled in (D), each sgRNA was used in a separate targeting experiment. Sites of DSB and PAM sites are indicated for each sgRNA, with DSB to point mutation distances highlighted. Mutations that introduce a novel restriction enzyme target sequence are also labelled.

**Supplementary Figure 4. MeCP2-mCherry positive cells lines as determined by immunofluorescence imaging**.

Neurons were differentiated until day 9 and then fixed and stained for neurofilament (NF), MAP2, mCherry and DAPI. Images are flattened z-stacks. Scale bar is 50 μm.
