# Supplementary figures and images for: Efficient and versatile CRISPR engineering of human neurons in culture to model neurological disorders
Source: Wellcome Open Res. 2016 Nov 15;1:13. [Version 1] doi: 10.12688/wellcomeopenres.10011.1 (PMC5146642; doi:10.12688/wellcomeopenres.10011.1)

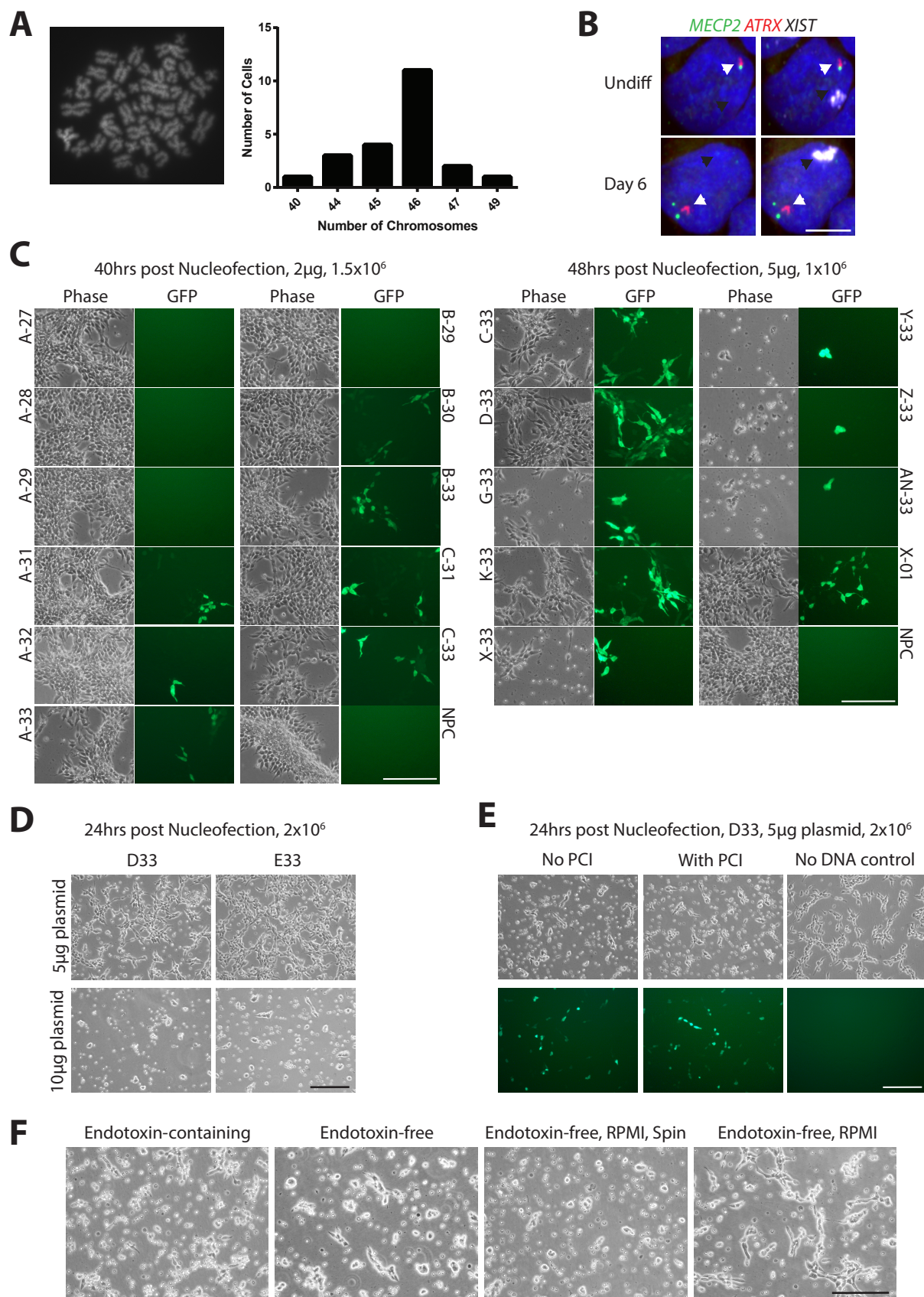

Supplement: Supplementary file 1 [file wellcomeopenres-1-10787-s0000.tgz › a3c81967-b094-4b35-9b3c-c064861ffed6.pdf]

**A**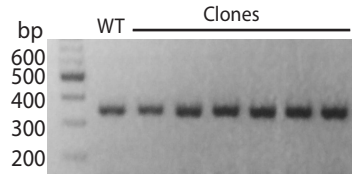**B**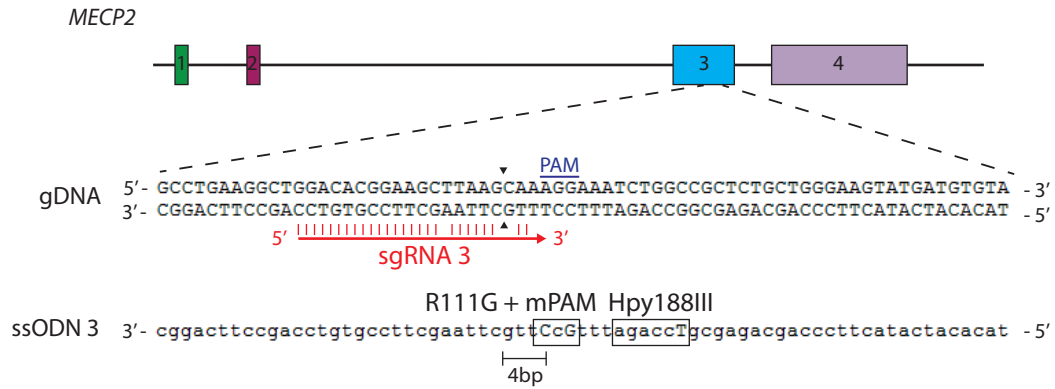**C**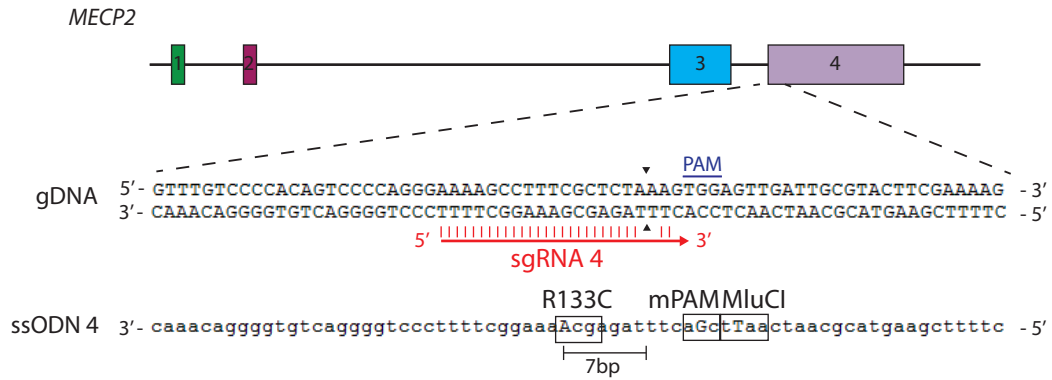**D**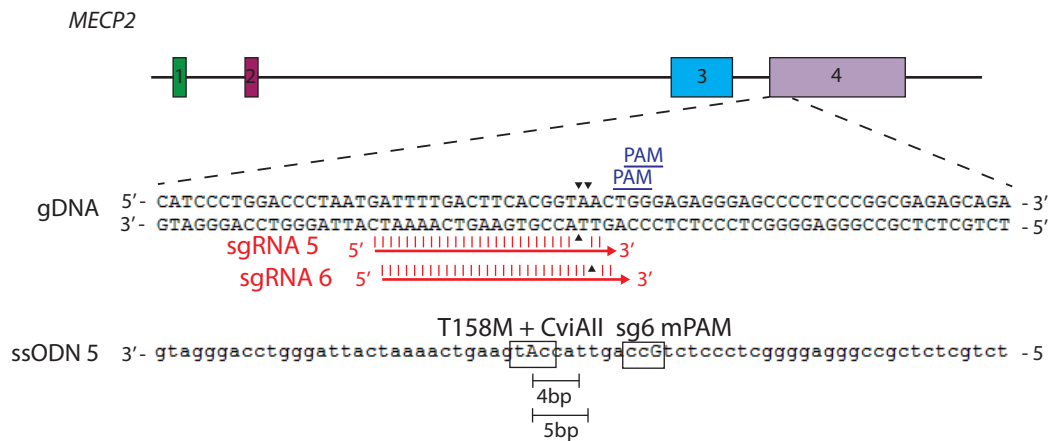

Supplement: Supplementary file 3 [file wellcomeopenres-1-10787-s0002.tgz › 205f0847-0406-4145-9131-435d9e70f1cc.pdf]

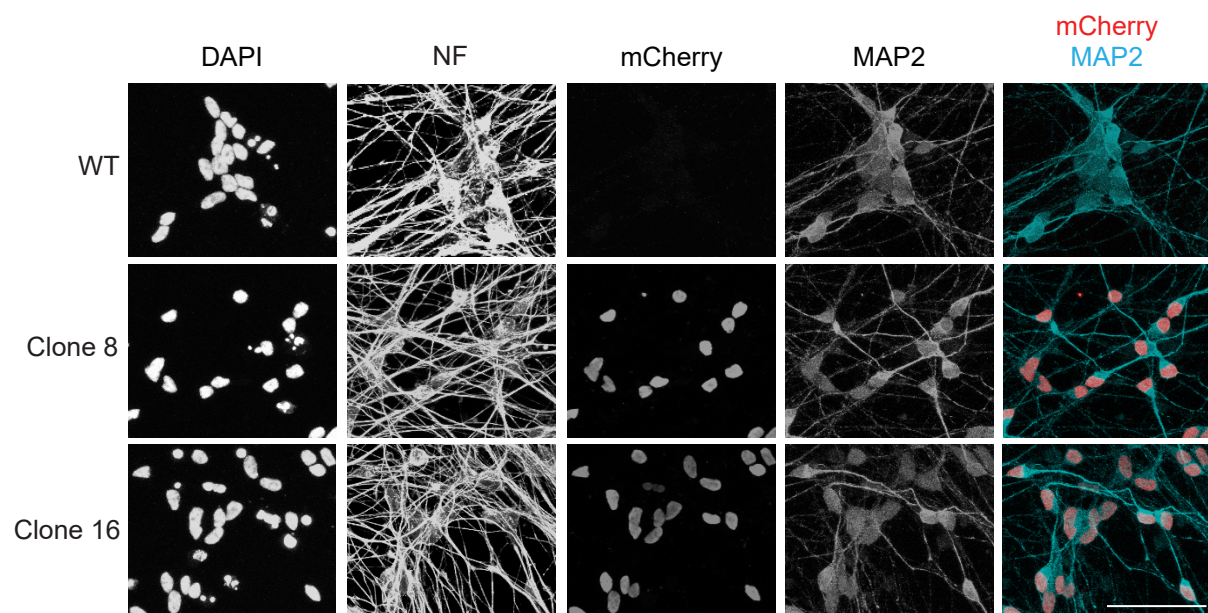

Supplement: Supplementary file 4 [file wellcomeopenres-1-10787-s0003.tgz › 8bb18d95-c00a-4598-bcde-12de5442d00a.pdf]
