# Supplementary material for: Efficient and versatile CRISPR engineering of human neurons in culture to model neurological disorders
Source: Wellcome Open Res. 2016 Nov 15;1:13. [Version 1] doi: 10.12688/wellcomeopenres.10011.1 (PMC5146642; doi:10.12688/wellcomeopenres.10011.1)
Supplement: Supplementary file 2 [file caca2023-d2ac-4ed5-ad02-633ac1a5d39a.pdf › caca2023-d2ac-4ed5-ad02-633ac1a5d39a.pdf]

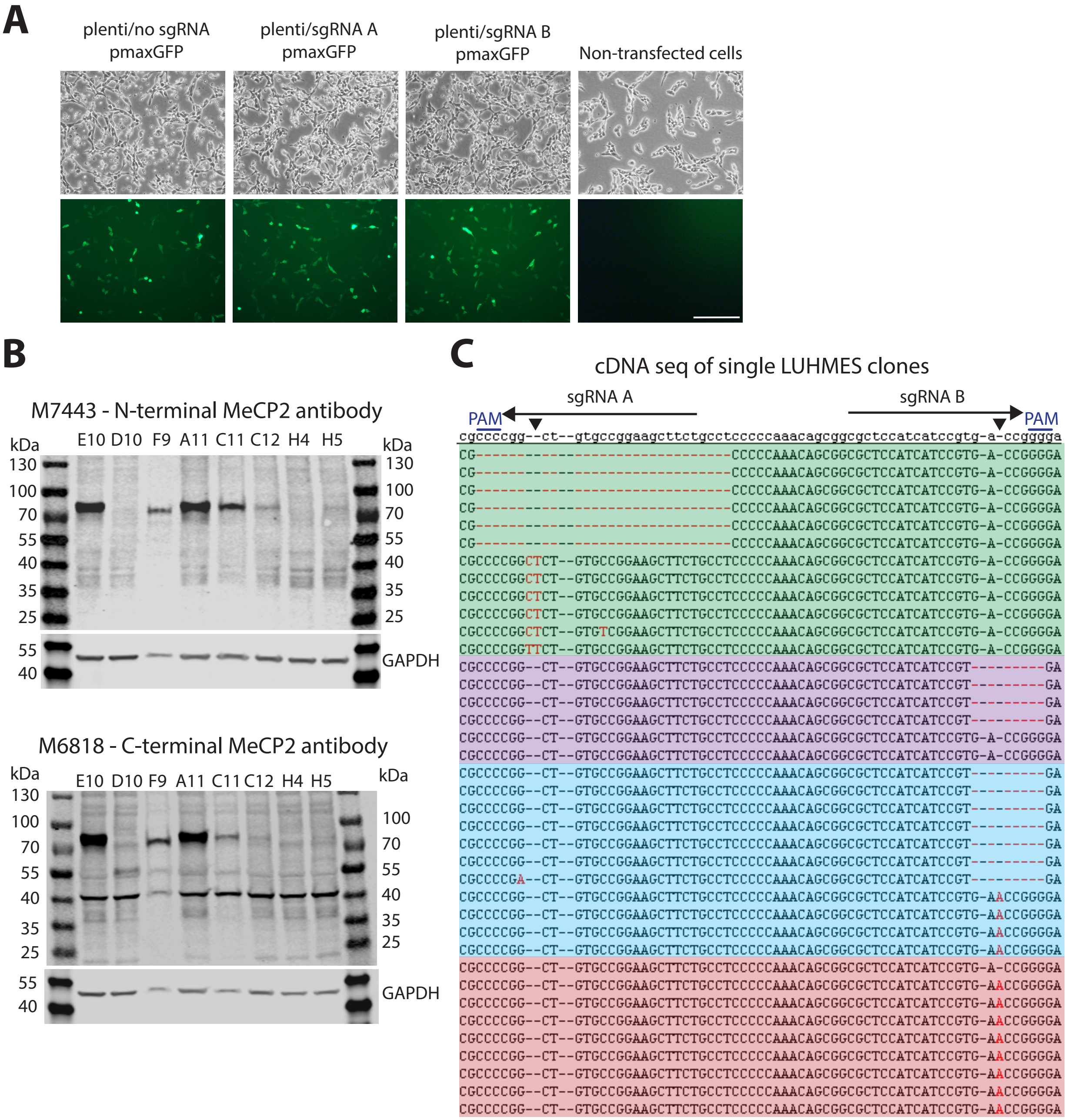
A
plenti/no sgRNA pmaxGFP
plenti/sgRNA A pmaxGFP
plenti/sgRNA B pmaxGFP
Non-transfected cells
B
M7443 - N-terminal MeCP2 antibody
kDa
E10 D10 F9 A11 C11 C12 H4 H5
130
100
70
55
40
35
25
GAPDH
M6818 - C-terminal MeCP2 antibody
kDa
E10 D10 F9 A11 C11 C12 H4 H5
130
100
70
55
40
35
25
GAPDH
C
cDNA seq of single LUHMES clones
sgRNA A
sgRNA B
PAM
PAM
