# Supplementary material for: Efficient and versatile CRISPR engineering of human neurons in culture to model neurological disorders
Source: Wellcome Open Res. 2016 Nov 15;1:13. [Version 1] doi: 10.12688/wellcomeopenres.10011.1 (PMC5146642; doi:10.12688/wellcomeopenres.10011.1)
Supplement: Peer review history

# Peer review: Referee response for version 1

**DOI:** 10.21956/wellcomeopenres.10787.r17639

**Published:** 2016-11-28

**Authors:** Patrik Brundin

**Affiliations:** 1 Center for Neurodegenerative Science, Van Andel Research Institute, Grand Rapids, MI, USA

This is a very clear and useful article describing protocols for the genetic modification of LUHMES cells. The paper is well written. The results are going to be helpful to the research community, not least because the authors also mention approaches that did not work. In view of the increased interest in using CRISPR engineering to modify human neurons and mimic disease in cell cultures, this paper will likely increase the number of laboratories that are interested in utilising LUHMES cells.

 There are only four minor concerns with the current edition of the manuscript:

1. Somewhere in the paper the authors should describe the approximate proportion of differentiated LUHMES neurons that express tyrosine hydroxylase (TH). This proportion has been reported to vary greatly between laboratories, and it would be helpful if this outcome is described briefly in the text (and possibly with a photo).
2. There is no description of what the different changes in phenotype the different genetic manipulations cause. Obviously, a detailed account is beyond the scope of the present manuscript, but a hint of the outcomes would be helpful. Alternatively, it should be clearly stated that this is the focus of another later paper.
3. The cells used in this paper (ATCC cat# CRL-2927, RRID CVCL_B057) are cited as a “kind gift from Dr Tanja Waldman”. ATCC stipulates “Please note that ATCC generally does not allow transfers of ATCC Materials or Progeny (“Unmodified Materials”). This is because ATCC Materials may be purchased directly from ATCC with limited disruption to research. […] Please note that there may be a fee for transferring Unmodified Materials to a third party as this falls outside of our general distribution practices.”
  (source: https://www.atcc.org/~/media/PDFs/ATCC%20MTA%20FAQs_2013.ashx).
  The authors should clarify briefly in the manuscript that they and Dr Waldman have obtained the appropriate permits that would make this gift comply with ATCC rules.
4. Since Wellcome Open Research is currently only indexed in PubMed Central, and not yet indexed in PubMed, the authors should promote the dissemination of this helpful manuscript via other channels (e.g. citing it in social media, indexed reviews and future papers).

I have read this submission. I believe that I have an appropriate level of expertise to confirm that it is of an acceptable scientific standard.

# Peer review: Referee response for version 1

**DOI:** 10.21956/wellcomeopenres.10787.r17631

**Published:** 2016-11-23

**Authors:** Tilmann Bürckstümmer, Rodrigo L dos Santos

**Affiliations:** 1 Horizon Genomics, Campus Vienna Biocenter, Vienna, Austria; 2 Horizon Discovery, Cambridge, UK

The authors present interesting data about the generation of new genetically engineered models for studying neurological disorders. In particular, the authors present optimized methods to generate KOs, KIs and reporter alleles in LUHMES cells. Such systems are of great relevance to establish direct links between disease-causing mutations and phenotypes.

 The manuscript is well written, has an important technical message, and is of acceptable scientific standard. I recommend its publication.

 However, there are some points that could be enhanced if some changes are made, or further clarification is provided. Mainly:

- The title should be changed to better describe the conclusions and cell type used in the study. “Efficient and veritable” could be changed to “optimized”, and “human neurons in culture” could be changed to “Immortalized Human Dopaminergic Neuronal Precursor Cells, LUHMES cells”
- The following sentence does not reflect recent findings - “Furthermore, there is still debate as to the robustness of the epigenome in iPSCs after reprogramming”. ( http://www.nature.com/nbt/journal/v33/n11/full/nbt.3388.html). Please update accordingly.
- Fig. 2D. The authors claim that KO1 clone is a homozygous KO clone, with both alleles harbouring a 9bp deletion. Although this is a fair assumption based on the data presented, this result could be based on technical artefact, such as PCR bias (only one the X chromosomes being amplified), or deletion of the primer binding sites due to big deletion generated after CRISPR cutting, or screening of insufficient bacterial clones to provide enough resolution. It would be clarifying if authors could provide further data to prove this point, like PCR using different primers pairs, further away from the cut site.
- Table 1. Taking into account that all positive clones in the “Number of KI clones” have been Sanger sequenced, it would be of relevance if the authors could discriminate how many of those are homozygous KI/KI, or heterozygous KI/WT or KI/indel. This information should also be added to the main text.
- In line with the comment above, similar information should be provided in the “Knock-in of a large tag into the endogenous MECP2 locus” section, mainly the discrimination of many clones were homozygous tag/tag, or heterozygous Tag/WT or Tag/indel.

 

 Overall, it is an important study by providing standardized methods for gene editing in LUHMES cells, which I believe is of important value for the scientific community.

We have read this submission. We believe that we have an appropriate level of expertise to confirm that it is of an acceptable scientific standard.
